# Comparison of Cardiovascular Disease Burden between Brazil and Denmark (1990–2021) Based on Global Burden of Disease: Availability of Universal Health System Is Not Enough

**DOI:** 10.5334/gh.1523

**Published:** 2026-02-20

**Authors:** Raphael Mendonça Guimarães, Isabella Tredler Zajdhaft, Laís Pimenta Ribeiro dos Santos, Leonardo Graever, Alixandre Vieira Araujo, Davidis Bechelli Correia da Silva, Eduarda Sanglar Sobreira, Sara Amaral Evangelista, Giulia Teixeira Monteiro Soares, Luiz Carlos Schrotke Pires, Viviane Gomes Parreira Dutra, Helena Dominguez

**Affiliations:** 1 National School of Public Health, Oswaldo Cruz Foundation, Rio de Janeiro, Brazil; 2 Medical Education Institute, Estácio de SáUniversity, Rio de Janeiro, Brazil; 3 Department of Biomedical Science, University of Copenhagen, Copenhagen, Denmark

**Keywords:** cardiovascular diseases, Global Burden of Disease, mortality, disability-adjusted life years, epidemiological transition

## Abstract

**Background:**

Cardiovascular diseases (CVDs) remain the leading cause of morbidity and mortality worldwide. High-income countries such as Denmark achieved sustained reductions, whereas middle-income countries such as Brazil continue to face high mortality and disability, reflecting distinct stages of the epidemiological transition.

**Objectives:**

To describe and compare temporal trends in age-standardized mortality and disability-adjusted life years (DALYs) due to CVD in Brazil and Denmark from 1990 to 2021, focusing on magnitude, stability, and inflection points.

**Methods:**

We conducted an ecological time-series study using Global Burden of Disease (GBD) 2021 estimates. Age-standardized mortality and DALY rates per 100,000 inhabitants were analyzed for Brazil and Denmark. Joinpoint regression identified changes in trends, estimating annual percentage change (APC) for each segment and average annual percentage change (AAPC) across the period. Analyses assumed Poisson distribution with robust variance and Monte Carlo permutation tests.

**Results:**

Between 1990 and 2021, CVD mortality declined by 53.9% in Brazil (AAPC: –2.4%; 95% CI: –2.7 to –2.1) and 69.7% in Denmark (AAPC: –3.6%; 95% CI: –3.9 to –3.2). DALY rates fell by 51.0% in Brazil (AAPC: –2.1%; 95% CI: –2.4 to –1.9) and 69.6% in Denmark (AAPC: –3.5%; 95% CI: –3.8 to –3.1). Denmark showed faster and more consistent reductions, with parallel declines in mortality and DALYs. In Brazil, mortality decreased more sharply than DALYs, suggesting persistent disability. Trend stability also differed: Brazil presented two joinpoints for each indicator, while Denmark exhibited only one.

**Conclusions:**

Both countries substantially reduced CVD burden, but disparities widened. Denmark’s experience reflects stable prevention, acute care, and rehabilitation strategies. Brazil’s progress, though significant, was uneven and limited by inequalities and slower declines in disability. Integrated policies are needed to ensure equitable and sustained reductions.

## Introduction

Cardiovascular diseases (CVDs) remain the leading cause of death worldwide, accounting for nearly one-third of all global deaths. In 2021, more than 19 million people died from CVD, with a substantial impact on years of life lost and disability (1). These conditions affect populations everywhere, but their distribution varies significantly across countries. High-income countries have experienced sustained declines in CVD mortality since the mid-20th century, while low- and middle-income countries continue to face a high burden, exposing inequalities in the epidemiological transition (2,3).

Comparing the burden of CVD in Brazil and Denmark is particularly relevant in this context. Brazil, a middle-income country of continental dimensions, combines rapid population aging, lifestyle changes, and persistent social inequalities. Despite progress, such as the expansion of the family health strategy and a strong reduction in smoking, the CVD burden remains high, marked by regional and socioeconomic disparities that reveal multiple stages of the epidemiological transition (4,5). Denmark, in contrast, is a high-income country with a consolidated universal health system that sharply reduced CVD mortality from the 1970s onward. Consistent prevention policies, dietary improvements, and therapeutic advances drove this decline (6). Today, Denmark mainly faces challenges of chronicity and aging, such as heart failure and atrial fibrillation.

This comparative analysis highlights not only socioeconomic and institutional contrasts but also the different results that universal health systems achieve when facing complex chronic conditions. Brazil’s Unified Health System (SUS) guarantees universal coverage despite financial limitations and deep regional inequalities. Denmark’s tax-funded and highly integrated system achieves greater homogeneity in cardiovascular health outcomes (7). By comparing both cases, we can assess how far universal systems succeed in ensuring equity and effectiveness under distinct contexts.

A central issue lies in the different models of epidemiological transition. In Brazil, the CVD burden grows while infectious diseases and external causes persist. Cerebrovascular diseases still weigh heavily, particularly in less developed regions, while ischemic heart disease and conditions linked to diabetes and obesity are increasingly prominent (8). In Denmark, by contrast, the burden shifted historically: ischemic heart disease and stroke declined, while chronic conditions associated with aging and survival became dominant (9). These differences in the composition of the CVD burden reflect distinct stages of the epidemiological transition and reinforce the need to interpret data within each national context.

By comparing Brazil and Denmark, this study also contributes to the global debate on health equity. The contrast between a middle-income country, marked by structural challenges and regional heterogeneity, and a high-income country, with consolidated preventive policies, forms a ‘natural experiment’ that illustrates how historical, demographic, and institutional conditions shape CVD burden. At the same time, the comparison shows that universal access, while necessary, is not sufficient to reduce CVD burden without consistent investments in prevention, risk-factor reduction, and efforts to address social inequalities (3,10).

The choice of these two countries also rests on the quality and availability of data. Both Brazil and Denmark are part of the Global Burden of Disease (GBD) estimates, which provide comparable time series from 1990 to 2021, disaggregated by age, sex, and CVD subtype. The methodological robustness of GBD, combined with consolidated national registries, ensures the feasibility and reliability of the analysis (1).

In this context, we hypothesized that Universal Health Coverage (UHC), while essential, does not by itself guarantee optimal and equitable reductions in CVD mortality and morbidity. By comparing two countries with long-standing and comprehensive UHC systems but distinct socioeconomic trajectories and health system organization, we aimed to test whether differences in the pace, stability, and composition of CVD burden reduction persist despite universal coverage. We further hypothesized that variations in health system performance—particularly in prevention, specialized care, and long-term management—would be reflected in divergent trends in mortality and disability-adjusted life years (DALYs).

In this sense, investigating temporal trends in CVD burden in Brazil and Denmark from 1990 to 2021 is thus relevant not only to describe epidemiological differences but also to understand how distinct development trajectories, public policies, and models of epidemiological transition shape the magnitude and composition of CVD. This analysis offers an opportunity to identify patterns, highlight lessons learned, and support evidence-based policies aimed at reducing the global burden of CVD. Based on this, the study aims to describe and compare trends in age-standardized CVD mortality rates and DALY rates in Brazil and Denmark from 1990 to 2021.

## Methods

### Study design

We conducted an ecological time-series study to examine the burden of CVDs in Brazil and Denmark from 1990 to 2021.

### Data source

We retrieved data from the GBD study, available through the open-access platform (https://www.healthdata.org/gbd/data-visualizations). The dataset covered the period from 1990 to 2021 for both countries. We relied on the International Classification of Diseases, 10th Revision (ICD-10) to define CVD causes. We used age-standardized mortality rates (ASMRs) and DALY rates for CVD. We calculated ASMRs for all ages and both sexes, standardized to the GBD standard population. Our analysis incorporated mortality and DALY estimates for the global population as well as for Brazil and Denmark. For international comparability, CVDs were defined according to the GBD study classification. This category includes ischemic heart disease, cerebrovascular disease (stroke), hypertensive heart disease, cardiomyopathy and myocarditis, atrial fibrillation and flutter, aortic aneurysm, peripheral artery disease, endocarditis, and other cardiovascular conditions as defined by GBD cause hierarchies. The use of the GBD framework ensures standardized case definitions, harmonized redistribution of ill-defined causes, and comparability of estimates across countries and over time.

### Time-series analysis

We applied segmented regression analysis (joinpoint regression) to identify significant changes in trends over time. This method assumes a linear trend between inflection points (joinpoints). Whenever the slope of the trend changed significantly from one segment to the next, we defined that moment as a joinpoint and initiated a new regression line. The joinpoint regression model, composed of several continuous linear phases, is widely used to describe temporal changes in epidemiological data. We specified the joinpoint model to fit the observed mortality and DALY trends for both countries.

(x_1_, y_1_), …, (x_n_, y_n_), where x_1_ ≤ … ≤ x_n_ without loss of generality, such as


\[
{\rm E}\left[{\rm y} \mid {\rm x}\right] = {{\beta}_{0}} + {{\beta}_{1}}{\rm x} + {\sum}_{{\rm j}=1}^{{\rm k}} {{\delta}_{{\rm j}}}{{\left({\rm x} - {{\tau}_{{\rm j}}}\right)}_{+}}, {\rm with} {{(a)}_{+}} = \max \left(a, 0\right),
\]

where τ_k_’s are the unknown joinpoints and a^+^ = a to a > 0 e 0 otherwise (11).

We performed the joinpoint regression analysis by treating CVD mortality and DALY rates as dependent variables. We centered the year variable in the regression models to simplify the interpretation of the intercept and to minimize collinearity between terms. To address temporal autocorrelation, we applied the *autocorrelated errors* option available in the Joinpoint Regression Program, which corrects for serial correlation in the residuals and provides unbiased estimates of the annual percentage change (APC) and its confidence intervals. To satisfy the assumption of homoscedasticity, we applied weighted least squares (WLS) in log-rates with reported standard errors. This method offers the advantage of identifying both the number and location of changes in the trend and estimating the APC for each period between inflection points (11,12).

To estimate the APC, we used the following model:


\[
\log \left({{{\rm Y}}_{{\rm x}}}\right) = {{\beta}_{0}} + {{\beta}_{1}}{\rm x},
\]

where log (Y_x_) is the natural logarithm from year x’ rate.

Then, the APC from year x to year x + 1 is:


\[
{\rm APC} = \frac{{{{\rm e}}^{{{\beta}_{0}} + {{\beta}_{1}}\left({\rm x}+1\right)}} - {{{\rm e}}^{{{\beta}_{0}} + {{\beta}_{1}}{\rm x}}}}{{{{\rm e}}^{{{\beta}_{0}} + {{\beta}_{1}}{\rm x}}} } \times 100 = \left({{{\rm e}}^{{{\beta}_{1}}}} - 1\right) \times 100.
\]

We defined the 95% confidence interval for APC as (APC_L_, APC_U_), where


\[
95 \% {\rm CI} = 100 \left[\exp \left( {{\beta}_{1}} \pm 1,96 {\rm SE}\left({{\beta}_{1}}\right)\right) - 1\right].
\]

We set the maximum number of joinpoints at four, given the 31-year observation period. The primary criterion for model selection was the Monte Carlo permutation test, with a two-sided significance level of 0.05. We used the Bayesian information criterion (BIC) as a secondary check to confirm model parsimony. All analyses were performed in the Joinpoint Regression Program, version 5.3.0 (Statistical Research and Applications Branch, National Cancer Institute, Bethesda, MD, USA, https://surveillance.cancer.gov/joinpoint/).

We tested additional joinpoints for the pandemic period (2020–2021), but they did not reach statistical significance. This result does not imply the absence of excess CVD mortality during COVID-19; rather, the short time frame of the pandemic years was not sufficient to generate detectable inflection points within the longer preexisting trend.

## Results

Between 1990 and 2021, Brazil and Denmark substantially reduced the burden of CVDs. However, Denmark achieved a greater reduction, which widened the gap between the two countries. This finding shows that although both countries made significant progress, the relative distance between them consistently increased (Table 1).

**Table 1:** ASMRs and DALY rates of cardiovascular diseases in Brazil and Denmark (1990–2021).


YEAR	ASMRS (95% CI)		DALY RATES (95% CI)	
	
	BRAZIL	DENMARK	BRAZIL	DENMARK

1990	333.67 (310.05–345.02)	314.09 (292.55–325.46)	7,427.69 (7,104.55–7,642.45)	5,910.72 (5,644.63–6,114.20)

1991	317.24 (294.41–328.37)	306.71 (285.11–318.12)	7,102.99 (6,791.62–7,323.54)	5,750.16 (5,491.72–5,938.72)

1992	311.76 (289.27–322.99)	304.34 (281.73–316.00)	7,003.32 (6,707.60–7,210.91)	5,670.50 (5,403.41–5,866.63)

1993	312.80 (289.67–323.69)	300.33 (277.87–311.20)	7,025.83 (6,735.36–7,240.00)	5,568.75 (5,299.13–5,752.51)

1994	302.62 (280.99–313.37)	287.11 (265.66–298.35)	6,820.74 (6,524.18–7,020.73)	5,331.81 (5,063.30–5,524.53)

1995	292.85 (271.03–303.28)	278.67 (256.70–290.16)	6,631.23 (6,337.78–6,840.28)	5,162.77 (4,876.64–5,352.37)

1996	285.64 (264.36–295.84)	264.96 (243.57–276.36)	6,486.61 (6,196.49–6,680.04)	4,924.52 (4,644.86–5,115.82)

1997	274.75 (254.06–285.08)	249.73 (229.15–260.86)	6,260.40 (5,974.69–6,460.18)	4,638.15 (4,373.43–4,827.20)

1998	269.27 (248.21–279.07)	235.33 (214.83–246.02)	6,160.73 (5,889.33–6,364.59)	4,389.01 (4,147.71–4,561.47)

1999	261.81 (241.35–271.55)	229.59 (209.98–240.00)	6,014.16 (5,740.53–6,213.85)	4,304.19 (4,069.96–4,485.17)

2000	252.01 (231.98–261.76)	216.34 (197.46–226.34)	5,804.89 (5,537.30–5,995.59)	4,014.97 (3,793.69–4,177.79)

2001	245.61 (226.52–255.47)	214.12 (195.06–224.18)	5,648.32 (5,381.51–5,839.94)	3,941.73 (3,709.61–4,111.92)

2002	240.15 (220.87–249.61)	208.01 (188.89–217.69)	5,493.70 (5,216.59–5,684.63)	3,828.31 (3,593.94–3,998.95)

2003	236.05 (217.17–245.34)	198.80 (179.76–208.82)	5,393.45 (5,129.01–5,570.20)	3,675.15 (3,425.73–3,845.66)

2004	231.88 (213.04–241.43)	186.98 (168.43–196.85)	5,302.57 (5,036.50–5,490.08)	3,469.99 (3,233.27–3,640.91)

2005	220.58 (201.72–229.98)	176.05 (158.72–185.53)	5,051.62 (4,778.63–5,227.02)	3,258.21 (3,035.21–3,419.97)

2006	217.38 (198.82–226.57)	169.33 (151.65–178.24)	4,974.77 (4,705.52–5,158.07)	3,145.42 (2,926.42–3,312.60)

2007	212.53 (194.37–221.62)	162.46 (144.90–171.48)	4,870.97 (4,611.13–5,053.92)	3,017.71 (2,808.61–3,175.37)

2008	208.68 (190.34–217.60)	153.86 (137.52–162.69)	4,789.23 (4,538.29–4,964.92)	2,853.67 (2,656.39–3,008.06)

2009	204.78 (186.64–213.63)	147.79 (131.51–156.17)	4,689.22 (4,430.40–4,866.66)	2,734.02 (2,534.93–2,883.64)

2010	200.37 (183.33–209.06)	141.30 (125.47–149.48)	4,580.91 (4,319.91–4,759.10)	2,610.05 (2,419.75–2,766.79)

2011	196.63 (179.13–205.21)	131.22 (115.83–139.66)	4,496.01 (4,239.45–4,674.66)	2,443.56 (2,259.50–2,595.43)

2012	189.62 (172.03–197.84)	125.47 (110.76–133.65)	4,346.16 (4,089.68–4,522.52)	2,336.15 (2,146.14–2,481.31)

2013	184.74 (168.43–193.24)	119.99 (105.78–127.49)	4,243.14 (4,005.89–4,421.16)	2,246.69 (2,067.65–2,389.64)

2014	179.53 (162.75–187.56)	114.74 (101.23–122.22)	4,129.48 (3,878.33–4,297.58)	2,157.70 (1,979.69–2,295.94)

2015	176.44 (159.83–184.38)	111.44 (98.10–118.69)	4,056.26 (3,819.36–4,227.63)	2,107.79 (1,929.78–2,245.22)

2016	176.38 (160.04–184.22)	108.77 (95.83–115.65)	4,060.43 (3,826.47–4,230.26)	2,063.48 (1,895.40–2,194.15)

2017	168.62 (153.18–176.19)	104.52 (92.24–111.07)	3,887.73 (3,651.96–4,050.30)	1,985.54 (1,830.01–2,118.01)

2018	163.37 (147.94–170.80)	102.54 (90.75–108.71)	3,778.31 (3,538.03–3,945.70)	1,945.69 (1,797.45–2,076.00)

2019	161.04 (146.25–168.47)	99.03 (87.38–105.33)	3,727.21 (3,502.00–3,902.49)	1,888.66 (1,742.20–2,017.99)

2020	159.56 (144.96–167.22)	95.85 (83.71–102.13)	3,713.92 (3,487.56–3,887.15)	1,830.53 (1,667.95–1,960.33)

2021	153.74 (140.12–161.93)	95.22 (82.46–101.93)	3,638.84 (3,415.03–3,812.26)	1,793.97 (1,637.65–1,926.71)


Source: GBD study (2025). Abbreviations: ASMRs, age-standardized mortality rates; DALY, disability-adjusted life years.

In 1990, Brazil recorded a cardiovascular mortality rate of 333.7 per 100,000 inhabitants, slightly higher than Denmark’s rate of 314.1 per 100,000 in the same year. By 2021, Brazil reduced its rate to 153.7 per 100,000, while Denmark reached 95.2 per 100,000. Both countries achieved substantial reductions, but the pace differed: Brazil reduced mortality by 53.9%, whereas Denmark reduced it by 69.7%. As a result, the Brazil:Denmark ratio increased from 1.06 in 1990 to 1.61 in 2021, and the relative difference grew from 5.9% to 38.1%.

**Figure 1:**
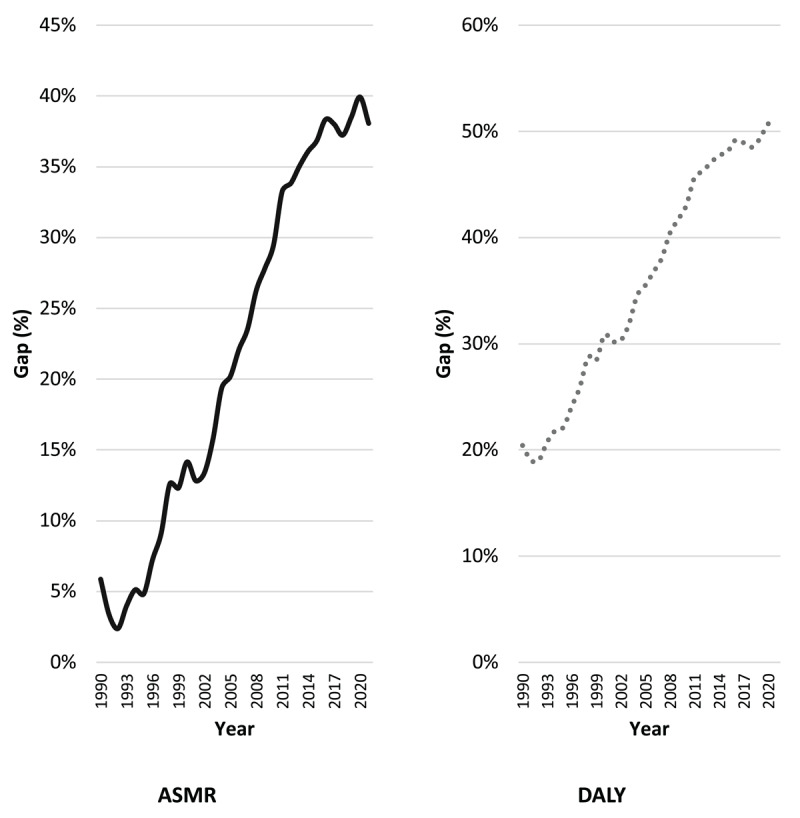
Gap between countries’ rates. Brazil and Denmark, 1990–2021. Source: GBD Study, 2025.

The DALY results followed the same pattern but revealed even sharper contrasts. In 1990, Brazil registered 7,427.7 DALYs per 100,000 inhabitants compared to Denmark’s 5,910.7, corresponding to a ratio of 1.26. In 2021, these rates fell to 3,638.8 in Brazil and 1,794.0 in Denmark, representing relative reductions of 51.0% and 69.6%, respectively. The Brazil:Denmark ratio, however, doubled to 2.03, and the relative difference expanded from 20.4% to 50.7% over the period (Figure 1).

The temporal trend analysis shows that both Brazil and Denmark consistently reduced cardiovascular mortality and DALY rates between 1990 and 2021 (Table 2). In Brazil, mortality decreased by an average of –2.4% per year (95% CI: –2.7 to –2.1), while DALYs fell slightly less, at –2.1% per year (95% CI: –2.4 to –1.9). This pattern indicates that although Brazil achieved major advances in reducing CVD deaths, the disability burden did not decline at the same pace, suggesting greater persistence of sequelae and chronicity among survivors.

**Table 2:** ASMRs and DALY rates of cardiovascular diseases in Brazil and Denmark (1990–2021).


RATE	PERIOD		APC (95% CI)	P VALUE

**ASMR**				

Brazil	1990	2005	–2.625 (–3.264 to –2.473)	<0.001*

	2005	2021	–2.276 (–2.416 to –1.706)	<0.001*

Denmark	1990	1993	–1.638 (–2.737 to 0.425)	0.219*

	1993	2003	–4.108 (–4.408 to –3.779)	<0.001*

	2003	2014	–4.767 (–5.772 to –4.558)	<0.001*

	2014	2021	–2.777 (–3.227 to –2.179)	<0.001*

**DALY**				

Brazil	1990	2014	–2.412* (–2.348 to –2.609)	0.004*

	2014	2021	–1.884* (–2.288 to –0.754)	0.028*

Denmark	1990	1993	–1.977* (–2.862 to –0.336)	0.039*

	1993	2004	–4.187* (–4.368 to –3.947)	<0.001*

	2004	2013	–4.770* (–5.554 to –4.558)	0.002*

	2013	2021	–2.756* (–3.049 to –2.396)	0.006*


Source: GBD study (2025). Abbreviations: APC, annual percentage change; ASMRs, age-standardized mortality rates; DALY, disability-adjusted life years.

In Denmark, the declines were sharper and more parallel: mortality decreased with an AAPC of –3.6% (95% CI: –3.9 to –3.2), and DALYs declined at –3.5% per year (95% CI: –3.8 to –3.1). The closeness of these values suggests that reductions in mortality are aligned with proportional reductions in disability burden. This outcome reflects a combination of effective prevention policies, equitable access to treatment, and consistent rehabilitation services. Therefore, in both countries, mortality and DALYs moved in the same direction, but Denmark’s faster decline highlights a more uniform and comprehensive process of reducing cardiovascular burden.

The differences between countries become clearer when analyzing the number and distribution of inflection points (joinpoints). In Brazil, we identified two joinpoints for mortality (1996 and 2010) and two for DALYs (1995 and 2008), suggesting a less stable trajectory with shifts in the pace of decline throughout the period. In Denmark, we found only one joinpoint for each indicator—in 2006 for mortality and 2007 for DALYs—indicating greater linearity and consistency in the downward trend. Moreover, the joinpoints did not occur in the same years in both countries, reinforcing the idea of distinct epidemiological and institutional trajectories. While Brazil’s changes took place earlier, in the second half of the 1990s and at the end of the 2000s, Denmark’s occurred mainly in the mid-2000s. Despite these differences, we did not observe any recent changes that reversed the declining pattern in either country.

## Discussion

The comparative analysis between Brazil and Denmark reveals clear trends: both nations reduced CVD mortality and DALY rates, but they did so with different speeds, stability, and consistency. This scenario calls for a broader reflection on structural, demographic, socioeconomic, and health policy factors that help explain these disparities.

In Brazil, cardiovascular mortality declined at a faster average annual rate than DALYs. This pattern suggests that reductions in deaths did not fully translate into comparable decreases in morbidity and disability associated with CVD. The persistence of relatively high DALY rates reflects sequelae, chronic conditions, and limited effectiveness of secondary prevention or rehabilitation strategies. These findings align with evidence showing that although relative risks of CVD mortality and DALYs decreased over the past decades, disability-related outcomes declined more slowly (13). In Denmark, by contrast, mortality and DALYs followed a parallel trajectory, with similar declines. This alignment highlights the country’s ability not only to prevent deaths but also to reduce or delay the onset and progression of CVD-related disabilities, likely through strong primary prevention, early diagnosis, and effective rehabilitation.

Denmark reduced both indicators more rapidly, as reflected by steeper negative AAPCs, which enhanced its comparative advantage over Brazil. The faster declines illustrate a more advanced stage of the cardiovascular epidemiological transition, characterized by fewer deaths and fewer long-term sequelae or chronic disabilities. This progress stems from systematic public health interventions, including anti-smoking policies, regulation of diet and alcohol, screening programs, and a strong primary care system. The broader social determinants of cardiovascular health also created fewer barriers to the impact of such policies (6,14). In Brazil, despite progress with tobacco control, expansion of primary care, and better access to therapies, persistent barriers—such as regional inequalities, uneven access, and rising metabolic risk factors—slowed DALY reductions. The literature supports this interpretation, showing that Brazil achieved significant reductions in age-standardized CVD mortality but only modest declines in the burden attributable to obesity, hypertension, and diabetes (2,15).

Brazil also displayed a higher number of joinpoints (two for mortality and two for DALYs), which indicates that its decline was not linear. Instead, mortality and DALY trends accelerated and slowed at different times, probably reflecting changes in policies, economic crises, or fluctuations in risk factors. Denmark, with only one joinpoint for each indicator, demonstrated greater temporal stability, with more linear and consistent declines. The timing of joinpoints also differed: Brazil’s occurred in the mid-1990s and again in the late 2000s, pointing to periods when interventions or structural shifts influenced outcomes. Denmark’s later joinpoints (mid-2000s) may reflect the exhaustion of earlier gains in cardiovascular health and the onset of more complex challenges, such as population aging and multimorbidity (16). Stability remains desirable because it reflects predictability and the ability to sustain policies; Brazil’s trajectory illustrates progress, but in phases of varying intensity.

Differences in AAPC reveal that the relative gap widened over time. Brazil started from higher levels and reduced them, but not fast enough to converge toward Denmark. This divergence has implications for global health equity: middle-income countries risk falling into a ‘disability trap’, where reductions in mortality do not align with equivalent reductions in years lived with functional limitations (2,12).

Several determinants explain these differences. Risk profiles diverge: in Brazil, smoking declined dramatically, but metabolic risk factors increased; in Denmark, reductions were more uniform across determinants (2,15). Demography also plays a role: Brazil’s rapid aging sustains high disability levels despite standardized declines, whereas Denmark’s prevention strategies mitigated the impact of aging. Health system organization further distinguishes the two countries: Denmark maintains universal coverage with consistent primary care, integrated management of comorbidities, and strong rehabilitation services, while Brazil faces persistent regional inequalities, uneven access, and chronic underfunding (17).

Both Brazil and Denmark structured their health systems on the principle of universality, but they differ sharply in financing, organization, and outcomes. Denmark finances its system primarily through general taxation, with decentralized management across five administrative regions responsible for hospital and primary care. Municipalities manage prevention, rehabilitation, and long-term care. This clear division of responsibilities ensures coordination and prevents service overlap (18). Brazil’s Unified Health System (SUS), created by the 1988 Constitution, is financed by federal, state, and municipal funds but has suffered from chronic underfunding, worsened by the 2016 Constitutional Amendment 95, which imposed a cap on public spending (19). The supply of health professionals also highlights inequality: Denmark has 4.38 physicians per 1,000 inhabitants, while Brazil has 2.14, with marked regional disparities (20). With 5.8 million inhabitants, Denmark ensures homogeneous coverage and equitable access. Brazil, with over 200 million people, faces stark contrasts between wealthy urban areas and remote rural regions, especially in the North and Northeast.

The policy implications are clear. In Brazil, mortality declined faster than DALYs, underscoring the need to invest not only in acute care but also in rehabilitation, secondary prevention, and management of sequelae. Brazil’s greater variability shows that progress has been inconsistent and dependent on external conditions, which emphasizes the importance of institutional continuity. Denmark might be used as an example on how a stable health system and sustained preventive policies can produce consistent, parallel declines in mortality and DALYs (5).

Several features of the Danish health care system likely contribute to its more consistent and balanced reductions in CVD mortality and disability. Denmark has long maintained comprehensive nationwide clinical registries that monitor cardiovascular care and quality metrics, facilitating continuous quality improvement and adherence to guideline-based therapy. For example, the Danish Atrial Fibrillation Registry has documented substantial increases in the use of echocardiography and oral anticoagulants among patients with atrial fibrillation, along with reductions in stroke risk, demonstrating how systematic monitoring can enhance management of high-risk conditions at the population level (21).

In addition, the Danish registry infrastructure supports research and health system planning across a range of cardiovascular procedures and interventions. The Western Denmark Heart Registry provides detailed, prospectively collected data on coronary angiographies, percutaneous coronary interventions, and other cardiac procedures, which support the evaluation of care quality and outcomes and inform improvements in treatment pathways (22). Likewise, the high quality of cardiovascular data from national patient registers enhances the validity of research and monitoring of treatment patterns and outcomes across regions (23). Collectively, these structural supports—national quality registries, linkage across health care data systems, and ongoing performance tracking—facilitate timely and equitable access to specialized care and secondary prevention, which may help explain the more uniform declines in both mortality and disability from CVD in Denmark relative to Brazil.

The widening gap between the two countries highlights the urgency for Brazil to accelerate reductions in cardiovascular disability. This challenge requires addressing social and regional determinants of health, expanding equitable access to specialized care, and strengthening population-level prevention strategies. Denmark’s experience shows that more consistent gains emerge when prevention, acute care, and rehabilitation work together. The comparison underscores not only different stages of the epidemiological transition but also the importance of robust health systems, stable policies, and long-term strategies to achieve balanced and sustainable reductions in cardiovascular burden (3,13,24).

A limitation of our analysis is that we did not explicitly model potential additional inflection points associated with the COVID-19 pandemic in 2020–2021. Although the joinpoint analysis did not detect reversals in the overall declining trends, excess mortality and delayed access to cardiovascular care during the pandemic may have temporarily altered the burden of CVD. This difference is noteworthy because the joinpoint method, while valuable for identifying changes in long-term trends, primarily serves as a hypothesis-generating tool. Future studies should investigate in greater depth how COVID-19-related disruptions may have influenced both short-term fluctuations and the persistence of long-term declines in cardiovascular mortality and disability. Another limitation is the reliance on age-standardized rates provided by the GBD study. While GBD applies a standardized methodology and reference population to ensure comparability across countries and over time, this approach may not fully capture context-specific demographic structures. Thus, although standardization facilitates cross-national comparisons, it may also mask heterogeneities within each country. Finally, it is important to emphasize that our findings derive from an ecological time-series design. Although the interpretation of trends considering national policies and health system characteristics provides plausible explanations, the study does not establish causal relationships. Rather, the results highlight temporal parallels between epidemiological trajectories and policy contexts. Making this distinction explicit reduces the risk of overinterpretation and underscores the need for complementary study designs to explore causal mechanisms.

An additional consideration concerns the composition of CVD subtypes. While we briefly introduced the contrasting weight of stroke versus ischemic heart disease, age-related shifts in the distribution of conditions such as heart failure and atrial fibrillation may partly explain why DALYs declined more slowly than mortality, especially in Brazil (25,26). This hypothesis remains important to test, but its verification requires disaggregated mortality and morbidity data by specific CVD causes, which are not available in the GBD dataset. Future analyses using local vital statistics or hospital records could clarify whether subtype-specific dynamics and age mixing drive the divergence between DALY and mortality trends.

As we have seen, Brazil and Denmark moved in the same direction of cardiovascular burden reduction, but they diverged in speed and stability, reflecting distinct stages of the epidemiological transition and differences in health system capacity. Denmark consolidated strong population-wide prevention policies and reorganized specialized care from the 1970s onward, achieving consistent parallel declines in mortality and DALYs (27). Brazil, despite important advances, still faces deep regional and social inequalities, with a greater impact on mortality than on disability (28). This pattern suggests that in middle-income countries with prolonged epidemiological transitions, such as Brazil, strengthening health system capacity to reduce sequelae and limitations is essential to ensure that longer survival does not translate into greater chronicity and disability. Integrated policies that combine prevention, equitable access to treatment, and rehabilitation remain urgent priorities to guarantee durable and balanced reductions in cardiovascular burden.

Finally, our findings suggest that providing UHC alone is not sufficient to achieve the maximum reduction in CVD mortality and morbidity. Although both Brazil and Denmark operate comprehensive universal health systems and have experienced substantial declines in CVD burden over the past three decades, the magnitude, stability, and balance between mortality and disability reduction differed markedly. These differences highlight the critical role of complementary policies beyond coverage, including sustained primary prevention, effective secondary prevention, timely access to specialized care, and integrated long-term management of chronic cardiovascular conditions. For policymakers and health program planners, this comparative evidence underscores that UHC must be accompanied by strong health system organization, continuity of care, and equity-oriented strategies to ensure that gains in survival translate into parallel reductions in disability and long-term cardiovascular burden.

## Data Accessibility Statement

The study used secondary data from the Global Burden of Disease Study. The dataset created from data extraction can be downloaded from https://github.com/raphaelfiocruz/CardiovascularDiseaseBRxDK.git.
